# Hidden Markov Trajectories of Early-Adolescent Media Overdependence and Machine Learning Prediction of High-Risk Maintenance from Early Childhood and Lower Elementary Predictors

**DOI:** 10.3390/bs15121725

**Published:** 2025-12-12

**Authors:** Eun-Kyoung Goh, Juyoun Kyun

**Affiliations:** 1 Human Life Research Center, Dong-A University, Busan 49315, Republic of Korea; ekgoh72@gmail.com; 2 Department of Early Childhood Education, Kaya University, Gimhae 50830, Republic of Korea

**Keywords:** media overdependence, digital addiction, problematic media use, early adolescence, early childhood/elementary predictors, high-risk maintenance, Hidden Markov Model trajectories, Machine Learning Prediction

## Abstract

Early adolescence is a sensitive period for digital media overdependence; however, persistent high-risk patterns remain poorly understood. Using data from the 2008 birth panel of the Panel Study on Korean Children (*n* = 1354), we examined predictors measured from early childhood to Grades 1–2 (2014–2016) and modeled digital media overdependence from Grades 3 to 6 (2017–2020). Hidden Markov Models (HMMs) were used to identify developmental trajectories, and machine learning models characterized risk signals using SHAP-informed feature importance. Five trajectories emerged, including one subgroup that maintained persistently high risk. The predictive model showed good discriminative performance (strong predictive performance [Receiver Operating Characteristic Area Under the Curve (ROC AUC) = 0.84]). Executive function difficulties in Grade 1 and their worsening through Grade 2 predicted an elevated risk, whereas longer or increasing sleep duration, stronger family interactions, and appropriate parental control were protective. In contrast, higher maternal parenting stress, greater overall media use time, and a larger proportion of game-centered media use functioned as risk factors. These findings identify modifiable early childhood and early elementary predictors of high-risk maintenance trajectories of media overdependence and may inform early screening and preventive interventions in families, schools, and communities.

## 1. Introduction

Today’s elementary school children are growing up in an environment in which digital technologies permeate virtually every aspect of their daily lives. Early adolescence, typically spanning Grades 3 through 6, represents a developmental transition period during which children experience significant increases in digital media exposure and access to personal devices. Survey research indicates that elementary school children are increasingly consuming online video- and game-based content and are gaining independent access to smartphones and tablets (Rideout & Robb, 2019; Rideout et al., 2022).

As peer interactions move further into digital environments (Subrahmanyam & Šmahel, 2011; Valkenburg & Peter, 2011), children may become particularly vulnerable to problematic or excessive media use. Although digital media can support learning and social connectedness (Canadian Paediatric Society, 2019), its excessive use has been associated with reduced self-regulation, sleep disruption, impaired daily functioning, and increased interpersonal conflict (OECD, 2024). International monitoring further underscores these concerns, with a WHO HBSC report showing an increase in problematic social media use from 7% in 2018 to 11% in 2022 (WHO Regional Office for Europe, 2024).

From a neurodevelopmental perspective, early adolescence is considered a critical period. The prefrontal systems underlying self-regulation are still maturing while environmental demands are increasing, making behavioral patterns particularly susceptible to consolidation (Crone & Konijn, 2018). However, much of the existing research relies on cross-sectional designs or single time-point predictors. As a result, there is limited longitudinal evidence regarding which children persistently follow high-risk trajectories of digital media overdependence and how these patterns evolve over time. Addressing this gap requires analytical approaches capable of capturing latent state changes and transitions, alongside theoretical frameworks that account for multidimensional childhood risks and protective factors.

Developmental cascade and risk accumulation models (Blair & Raver, 2015; Masten & Cicchetti, 2010) provide a strong conceptual foundation for anticipating stable, high-risk pathways. These models posit that early vulnerabilities, such as executive function difficulties, sleep problems, parental stress, and reduced parent–child interaction, interact and accumulate across development, shaping long-term self-regulation trajectories. From this perspective, persistent media overdependence may emerge when early risk processes compound and reinforce one another over time. Thus, this framework offers a compelling rationale for modeling longitudinal transitions in risk states rather than focusing solely on single time-point associations.

The predictors examined in this study were selected for their theoretical relevance and empirical support within the developmental, self-regulation, and media use frameworks (Baumeister & Heatherton, 1996; Brand et al., 2014; Y. Chen et al., 2024; Diamond, 2013; Domoff et al., 2020; Fitzpatrick et al., 2025; Linebarger et al., 2014; McDaniel & Radesky, 2018). These factors have consistently emerged as core mechanisms underlying problematic media use and represent modifiable targets for early intervention (Linebarger & Vaala, 2010; Madigan et al., 2020; Radesky et al., 2014). Because the developmental pathways identified through the Hidden Markov Models (HMM) reflect latent trajectories of self-regulatory development rather than static clusters, it is theoretically meaningful to hypothesize that early regulatory capacities and environmental conditions shape whether children remain in or transition out of high-risk pathways.

Executive function (EF) has been established as a central mechanism of self-regulation and predictor of impulsivity, attentional dysregulation, and problematic media use (Diamond, 2013; Fitzpatrick et al., 2025). Sleep provides a biobehavioral foundation for emotional and behavioral regulation, and early sleep problems predict later internalizing and externalizing difficulties (Blacher et al., 2024; Quach et al., 2018). Furthermore, screen time has been consistently linked to reduced sleep duration and later bedtime (Bauducco et al., 2024; Brosnan et al., 2024; Y. Chen et al., 2024). Family factors, including parenting stress, parent–child interaction, and early media exposure, further shape children’s self-regulation and subsequent media-use patterns (Madigan et al., 2020; McDaniel & Radesky, 2018). Given that these domains interact and accumulate over time, identifying how early childhood factors shape trajectories of digital media overdependence is an essential developmental question.

From a methodological standpoint, this study applies the HMM to estimate latent states and transition probabilities (Rabiner, 1989). The HMM is well-suited for examining media-use trajectories because it does not require predefined latent profiles (Bartolucci et al., 2012) and can identify data-driven state patterns when the underlying structure is unknown. By contrast, latent transition analysis requires predefined latent profiles, making HMM a more appropriate choice when the latent-state structure has not yet been empirically established. Furthermore, machine learning approaches, including SHAP-based interpretation, enable the examination of nonlinear relationships among multidimensional early predictors, which is an improvement over prior work that has often relied on limited predictor sets or single-time-point measures. Together, these analytical approaches strengthen explanatory precision and support the earlier identification of children at elevated risk.

Finally, this study draws on the interactional theory of problematic childhood media use (IT-CPMU; Domoff et al., 2020). This theory posits that self-regulatory vulnerabilities interact with family and contextual factors, thereby reinforcing problematic media use patterns over time. The high-risk maintenance pathways identified in this study reflected these interactional processes and underscored the need for developmentally timed interventions.

Building on this foundation, this study investigated how multidimensional early childhood and early elementary school factors shape the developmental trajectories of digital media overdependence in early adolescence. Using long-term longitudinal data, we identified HMM-based developmental pathways and detected early risk signals using machine learning to inform prevention and intervention strategies across family, school, and community settings. Accordingly, we addressed the following two research questions:

1. When distinguishing the developmental trajectories of digital media overdependence among early adolescents using HMM, can we statistically identify a subgroup with a persistently high-risk status that is distinct from other trajectories?
2. Which early and middle childhood factors predict a high-risk maintenance trajectory of digital media overdependence in early adolescence?

## 2. Methods

### 2.1. Participants and Data Source

We analyzed secondary data from the Panel Study on Korean Children (PSKC), a nationally representative panel survey of Korean children and their families that initially recruited families of children born in 2008, and followed them annually thereafter. In the present study, we used four consecutive assessments from wave 10 (age 9) to wave 13 (age 12). Children who participated in all four waves were included (*n* = 1354), comprising of 686 boys (50.66%) and 668 girls (49.34%). Sex was coded as 1 for boys and 2 for girls, with boys used as the reference category in the subsequent analyses. Using an HMM classification of media overdependence trajectories across waves, 188 children (13.9%) were identified as the persistent high-risk group, and 1166 (86.1%) as the non-high-risk group. The Institutional Review Board of Dong-A University exempted the study (exemption no. 2-1040709-AB-N-01-202507-HR-048-01; determination date: 17 July 2025).

### 2.2. Measures

#### 2.2.1. Outcome: Media Overdependence (Scoring and Risk Classification)

At each wave (w10–w13), media overdependence was assessed using a standardized instrument comprising of three subscales: self-regulation difficulty (F1), daily life interference (F3), and control failure (F4). The internal consistency was high across the waves (Cronbach’s α = 0.840, 0.854, 0.864, and 0.855, respectively). The instrument and all risk thresholds were based on the nationally validated Korean Smartphone and Media Overdependence Scale developed by the National Information Society Agency is widely used for population monitoring and clinical screening (Yang & Kim, 2021). No new cut-off values were introduced in the present study.

Risk categories were assigned using validated concurrent thresholds. Children were classified as general users when the total score was ≤27 and subscale scores satisfied F1 ≤ 12, F3 ≤ 10, and F4 ≤ 9. They were classified as potential-risk users when the total score was 28–29 or subscale scores met F1 ≥ 13, F3 ≥ 11, and F4 ≥ 10, provided that high-risk criteria were not met. High-risk users were defined as those with a total score ≥30 or subscale scores satisfying F1 ≥ 14, F3 ≥ 12, and F4 ≥ 11. Thresholds were applied concurrently; category assignment required meeting both the total score range and the corresponding subscale cutoffs. In cases of overlap, the classification defaulted to the high-risk category.

These wave-specific classifications were used as the observed emissions for the HMM, which estimated the latent states (normal, at-risk, and high-risk) and transitions across the waves. For machine learning analyses, we constructed a binary outcome variable coded 1 for children who were classified as high risk in all four waves (persistent high risk) and 0 otherwise.

#### 2.2.2. Predictor Variables

Predictor variables were drawn from waves 7 to 10 and spanned the demographic, child, and parent–family domains. The demographic variables included the child’s sex and age, maternal and paternal age, proportion of parental university education, and monthly household income. Child-related factors included externalizing problems (cbt_expT_c7), EF difficulties (exf_c8), school adjustment (pScl_c8; single item), sleep duration (sleepTime_c7), overall media use time (midUseTime_c8), proportions of gaming- and learning-oriented use (midUse_game_c9 and midUse_learn_c9), and longitudinal change indices.

Parent/family factors included maternal parenting stress (prs_m7), maternal–family interaction (fli_m7), maternal controlling parenting (crs_ct_m7), paternal controlling parenting (crs_ct_f7), and the corresponding residualized change scores. Multi-item scales demonstrated acceptable reliability (Cronbach’s α ≥ 0.70): exf_c8 = 0.944, prs_m7 = 0.881, fli_m7 = 0.912, crs_ct_m7 = 0.737, and crs_ct_f7 = 0.786. All continuous predictors were standardized using z-scores.

The residualized change variables (prefixed with “change_”) were computed as the residuals from regressing later-wave scores on earlier-wave scores. These values represented the net change from the baseline. Positive scores indicate increases greater than expected given the baseline, whereas negative scores indicate below-expected increases or relative declines. When the baseline and residualized change variables were included simultaneously in the models, the variance inflation factors were below 2.5, suggesting that multicollinearity was not a substantive concern.

Information on variable coding is provided in Supplementary Table S1. The maximum item-level missingness for any predictor was 16%, with all missingness rates being below 20%. Pairwise correlations among the predictors were below 0.50, and the full correlation matrix is shown in Supplementary Figure S1.

### 2.3. Missing Data Handling

Missingness was summarized at both the wave and predictor levels. Wave-level missingness across waves 10–13 ranged from 0.05% to 6.1%, and the maximum item-level missingness among predictors was 16% (all < 20%); detailed percentages for each variable are provided in Supplementary Table S2. Missing predictor values were imputed using multivariate imputation with chained equations and predictive mean matching, generating 20 datasets, which were pooled according to Rubin’s rules. The imputation model incorporated all predictors alongside conceptually related auxiliary variables (e.g., concurrent indicators of sleep, parenting stress, and family interaction) to strengthen the plausibility of the missing-at-random assumption. The convergence of the MICE algorithm was evaluated using trace plots to examine the stability of the chain means across 50 iterations, which confirmed stable mixing with no systematic trends (see Supplementary Figure S2). In addition, the density plots show comparable distribution shapes between the observed and imputed values, supporting the adequacy of the imputation process (see Supplementary Figure S3).

To avoid circularity and artificial stabilization of the state process, the HMM-derived latent states and trajectory labels were excluded from the imputation model. As a result, the HMM estimation was conducted separately using the original observed risk categories. Media-overdependence outcomes were not imputed because the risk labels were determined using HMM.

Missing data were imputed using the MICE package (van Buuren & Groothuis-Oudshoorn, 2011), and missing-data patterns were visualized using *VIM* (Kowarik & Templ, 2016).

### 2.4. Analytical Strategy

The analysis is conducted in three stages. First, we generated descriptive statistics and conducted bivariate comparisons (*t* tests and *x*^2^ tests) between the persistent high-risk and non-high-risk groups, with internal consistency assessed using Cronbach’s α. Second, we estimated the HMMs to identify the latent states of media overdependence and their transition probabilities across waves. Model fit indices-guided state selection, and Viterbi decoding were used to assign the most likely latent state sequence for each child. Third, we constructed supervised machine learning models to predict persistent high-risk membership based on baseline predictors.

These steps involved data wrangling and visualization using the *tidyverse* suite (Wickham, 2016; Wickham et al., 2019), including *dplyr*, *tidyr*, and *ggplot2*. The machine learning component was implemented within a predictive rather than a causal inference framework to support early screening and risk identification, rather than to estimate causal effects.

### 2.5. Hidden Markov Modeling

Wave-to-wave changes in the observed risk categories (general, potential, and high-risk) were modeled using a time-homogeneous first-order HMM. Candidate models with two to four latent states were fitted and compared based on the log-likelihood, Akaike Information Criterion (AIC), Bayesian Information Criterion (BIC), entropy, and interpretability to determine the optimal specification. Model parameters, including initial state probabilities, transition matrices, and state-dependent emission probabilities, were estimated using the EM (Baum–Welch) algorithm with multiple random starting values to avoid local optima, and individual state sequences and posterior probabilities were derived using Viterbi decoding. When the transition probabilities could not be extracted directly from the fitted model, they were estimated from the iterbi-decoded transitions as a pre-specified fallback. Classification uncertainty was summarized using posterior probabilities. For downstream machine learning analyses, a persistent binary high-risk label was created, defined as being decoded as high risk for all four waves. All HMM analyses were performed in R using the depmixS4 package (Visser & Speekenbrink, 2010). Although the wave-specific classifications were based on validated cutoff criteria, these thresholds can be sensitive to short-term fluctuations and may over discretize the risk. HMMs refine these classifications by incorporating temporal continuity and probabilistic state decoding, thereby reducing noise from single-wave misclassification and providing more developmentally meaningful estimates of persistent or changing media use risk states over time. Thus, HMM-derived latent states serve as conceptually sound and methodologically robust outcomes for predicting developmental trajectories of media overdependence risk.

### 2.6. Machine Learning Modeling

An extreme gradient boosting (XGBoost) classifier was implemented in R (version 4.3.2) using the XGBoost package (T. Chen & Guestrin, 2016) within the care framework (Kuhn, 2008). Predictors were drawn from waves 7 to 9, spanning early childhood through Grade 2. The binary outcome, “persistent high risk,” was derived from HMM-decoded states across waves 10–13. This temporal ordering ensured that all the predictors preceded the outcome and prevented information leakage.

The dataset was divided into training (70%), validation (15%), and test (15%) sets using stratified sampling at the child ID level. Because the PSKC included only one target child per family, no sibling clustering was present. To address class imbalance, the training set was upsampled prior to model selection. Oversampling was applied only to the training set, whereas the original class distributions of the validation and test sets were retained to avoid leakage. Cross-validation employed a grouped 3-fold procedure (groupKFold), using the area under the ROC AUC as the optimization criterion. This approach separates model tuning in the oversampled folds from the threshold selection in the validation set, with a final evaluation conducted on the untouched test set.

The hyperparameters were tuned using a grid search, and the tuning ranges are summarized in Supplementary Table S7. The preprocessing included median imputation, zero-variance filtering, centering, and scaling. A decision threshold was selected for the validation set using Youden’s index and the final predictive performance was evaluated using the independent test set. Discrimination was summarized using ROC AUC (DeLong confidence intervals) and Precision–Recall Area Under the Curve (PR AUC) (bootstrap confidence intervals), computed via *pROC* (Robin et al., 2011) and *PRROC* (Grau et al., 2015), and comparative performance across classifiers (ROC AUC, F1, PR AUC) is reported in Supplementary Table S6.

Calibration was assessed using the Brier score, Hosmer–Lemeshow test, and reliability curves. The Hosmer–Lemeshow test was performed using the ResourceSelection package (Lele et al., 2019), and calibration binning for reliability diagrams was computed using the data table package (Dowle & Srinivasan, 2022). The expected calibration error (ECE) and maximum calibration error (MCE) were estimated using bootstrap confidence intervals. Threshold-dependent metrics, including sensitivity, specificity, positive predictive value (PPV), negative predictive value (NPV), accuracy, and F1-score, were computed for the test set.

Predictor contributions were examined using model-based feature importance (via Caret’s varImp), and SHAP values were computed using the SHAPforxgboost package (Liu, 2020). These SHAP values provide a predictive rather than a causal interpretation of the model behavior.

## 3. Results

### 3.1. Longitudinal Trends in Measured Media Overdependence

As shown in Table 1, across four annual waves (w10–w13), the total media-overdependence score increased from *M* = 23.59 (*SD* = 5.94) at w10 to *M* = 27.24 (*SD* = 6.52) at w13, and this change was statistically significant (*F* = 1925.98, *p* < 0.001).

### 3.2. HMM Analysis of Measured States

To characterize the latent transitions, we fitted the HMMs to two, three, and four states (Table 2). All models converged. The three-state model provided an optimal balance between fit and parsimony (AIC = 7740.34; BIC = 7832.09). The classification entropy was 0.562, indicating moderate certainty. Because the HMM entropy reflects posterior probability dispersion rather than static accuracy, this value suggests a partial overlap across adjacent states for some children. The emission probabilities of each state are listed in Supplementary Table S4. Additionally, bootstrap-based 95% confidence intervals for the latent state transition probabilities are reported in Supplementary Table S5 to quantify the uncertainty in the state dynamics.

The transition matrix (Table 3) revealed strong stability in the high-risk state (0.897). The at-risk state transitioned toward both the high-risk (0.321) and normal (0.118) states. The normal state remained stable (0.750) but demonstrated movement toward the at-risk state (0.250). These patterns suggest that high-risk status tends to persist, at-risk status exhibits bidirectional movement, and normal status generally remains unchanged. Bootstrap-based 95% confidence intervals for key transitions (e.g., Normal, At-risk, High-risk, High-risk) showed reasonably narrow ranges, supporting the stability of these estimates. The detailed classification criteria, emission probabilities, and transition probability estimates with confidence intervals are reported in Supplementary Tables S3–S5.

It is important to distinguish between “states” and “trajectories.” The HMM identified three time-specific latent states, whereas the five trajectories reflected multiyear patterns in the decoded sequences. Thus, the trajectories represent longitudinal profiles rather than additional latent states.

### 3.3. HMM-Derived Developmental Trajectories

**Figure 1:**
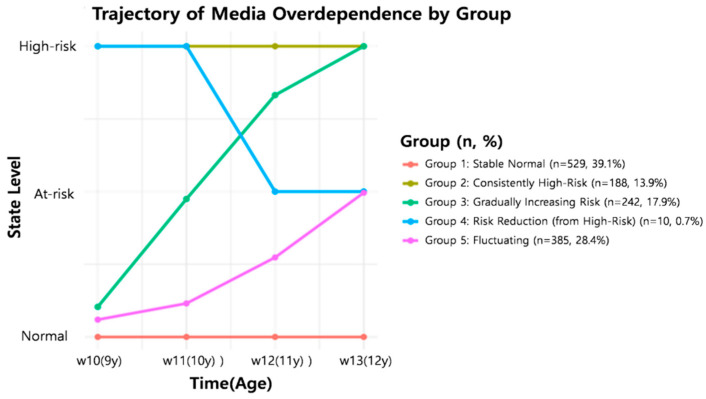
Five developmental trajectories.

Posterior decoding was used to identify five trajectory groups (Figure 1). The Stable Normal group accounted for 39.1% of the analytical sample, the Stable High-Risk group for 13.9%, the Gradually Increasing Risk group for 17.9%, the Risk Reduction (from high risk) group for 0.7%, and the fluctuating risk group for 28.4%. These proportions indicate that, within the present analytical sample, the largest share followed a consistently normal pattern, a smaller share maintained a consistently high-risk pattern, and the remaining groups showed increasing, decreasing, or fluctuating patterns over time (Supplementary Table S3).

The risk reduction group should be interpreted descriptively rather than inferentially because its size was extremely small (0.7%). This rare pattern likely reflects the small number of children whose observed transitions follow a decreasing risk sequence; however, classification uncertainty and potential misclassification cannot be ruled out. Accordingly, we refrained from drawing strong theoretical or policy conclusions from this subgroup. Nonetheless, the presence of a small decreasing risk pattern underscores that the developmental trajectories of media overdependence are heterogeneous rather than strictly uniform.

A moderate entropy indicates that minor-state misassignments may have occurred. However, as the persistent high-risk group required a high-risk status for every wave, it was less sensitive to uncertainty in any single wave.

### 3.4. Descriptive and Bivariate Results for Machine Learning Predictors

For the machine learning analysis, the persistent high-risk outcome was defined as being classified as high risk for all four waves, corresponding to a Stable High-risk trajectory. The analytical sample included 188 participants in the high-risk ML group and 1166 participants in the non-high-risk group (Table 4).

In the sociodemographic comparisons, the ML high-risk group contained a higher proportion of boys (62.8 percent vs. 48.7 percent, *p* < 0.001) and a lower proportion of mothers with college or higher education (64.4 percent vs. 74.6 percent, *p* = 0.004).

Persistently high-risk children showed higher levels of preschool-externalizing behaviors (*p* < 0.001), shorter sleep duration (*p* < 0.001), and poorer executive functioning in Grade 1 (*p* < 0.001), with an additional decline through Grade 3 (*p* < 0.001). School adjustment was lower (*p* < 0.001) and worsened over time (*p* = 0.037). Media engagement was higher in Grade 1 (*p* < 0.001) and increased over time (*p* = 0.002). The share of gaming in total media use was larger (*p* < 0.001), whereas the share of learning-oriented media use was smaller (*p* < 0.001).

Regarding parental and family factors, mothers reported higher parenting stress (*p* < 0.001), with an increasing trend over time (*p* < 0.001). Maternal–family interaction was lower (*p* < 0.001) and declined more steeply (*p* < 0.001). Both maternal and paternal parenting controls increased significantly in the high-risk ML group (*p* < 0.05).

These comparisons indicate that within the data analyzed in this study, the ML high-risk group differed from the non-high-risk group across the sociodemographic, child, and parental domains, as summarized in Table 4.

### 3.5. Machine Learning Model Performance

**Figure 2:**
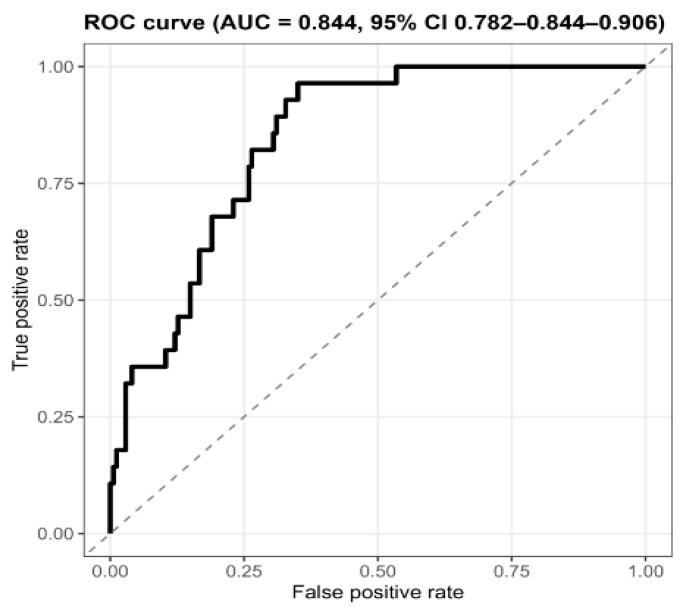
Receiver operating characteristic (ROC) curve for the test dataset.

On the independent test set, the XGBoost classifier demonstrated strong discrimination for persistent high risk (ROC AUC = 0.84, 95% CI 0.78–0.91; DeLong), which is generally considered good performance (ROC AUC ≥ 0.80) (Table 5). Figure 2 shows the ROC curve for the test dataset, illustrating the overall model discrimination. The details of the model configuration are listed in Supplementary Table S7 (XGBoost hyperparameters). At the prespecified decision threshold of 0.2954, the confusion matrix comprised 23 true positives, 51 false positives, 123 true negatives, and 5 false negatives (see Supplementary Table S8: Confusion matrix and basic classification summary). Threshold-dependent metrics at this operating point were sensitivity 0.82 (95% CI, 0.63–0.94), specificity 0.71 (95% CI, 0.63–0.77), PPV 0.31 (95% CI, 0.21–0.43), NPV 0.96 (95% CI, 0.91–0.99), F1-score 0.45 (95% CI, 0.33–0.56), and accuracy 0.72 (95% CI, 0.66–0.78) (Table 5). Given the observed prevalence of 0.14, a PPV of 0.31 represents enrichment over the baseline at this threshold, and a NPV of 0.96 indicates a low false-negative rate among non-high-risk predictions. Calibration was acceptable by summary indices: Brier score 0.13, ECE 0.15 (95% CI, 0.11–0.19), and MCE 0.57 (95% CI, 0.44–0.82) (Table 5). The close agreement between the cross-validated performance on the training data and the performance on the independent test set suggests that substantial overfitting is unlikely. Confidence intervals were obtained using a 2000-sample bootstrap, except for the ROC AUC, which used DeLong’s method. Under class imbalance (13.9% positives), the precision–recall (PR) curve yielded an PR AUC of 0.48 (95% CI, 0.29–0.65), illustrating stable precision–recall trade-offs across thresholds; the full PR curve is presented in Supplementary Figure S4 (class imbalance performance).

### 3.6. Key Predictors Identified by the Machine Learning Model

**Figure 3:**
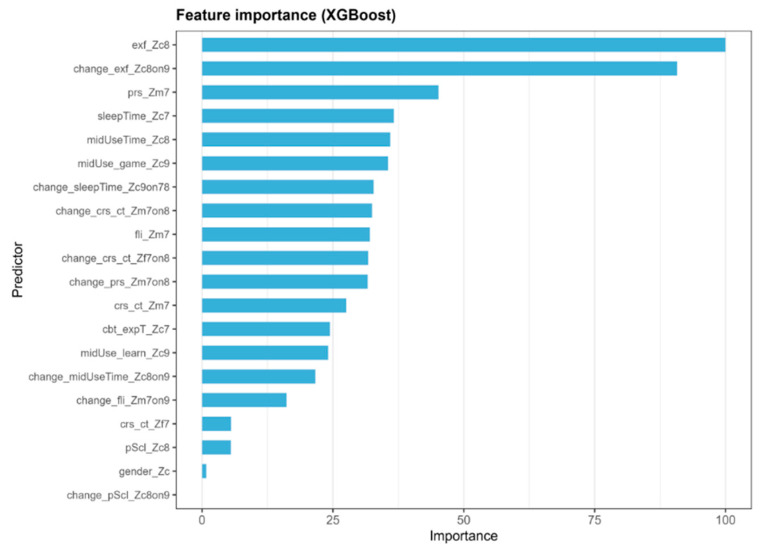
Feature importance plot.

**Figure 4:**
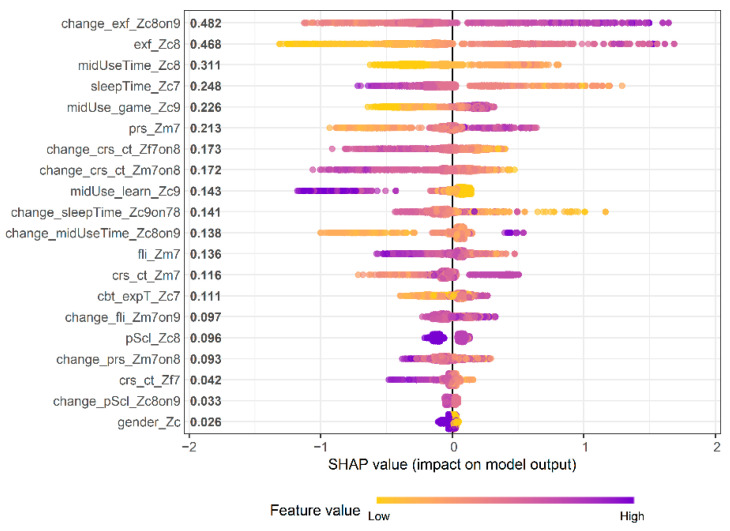
SHAP summary plot. *Notes for interpretation.* Feature importance and SHAP reflect model-specific associations and do not imply causality; SHAP magnitudes are on the model’s log-odds scale and are not directly comparable to standardized effect sizes.

The feature importance and SHAP analyses converged on a consistent set of predictors for the classification of persistently high risks (Figure 3 and Figure 4). In the XGBoost feature importance ranking, EF difficulties in Grade 1 (exf_Zc8) and their residualized change between Grades 2 and 3 (change_exf_Zc8on9) were the two most influential variables, indicating that both lower baseline EF levels and subsequent declines, adjusted for prior levels, were associated with a higher model-predicted probability of persistent high risk. Additional contributors with substantial importance included maternal parenting stress in preschool (prs_Zm7), preschool sleep duration (sleepTime_Zc7), overall media use time in Grade 1 (midUseTime_Zc8), and the proportion of gaming within the total media use in Grade 2 (midUse_game_Zc9). Other notable predictors included residualized changes in sleep duration (change_sleepTime_Zc9on78), maternal and paternal parenting controls (change_crs_ct_Zm7on8; change_crs_ct_Zf7on8), and maternal parenting stress (change_prs_Zm7on8). Lower-than-expected sleep duration or a greater-than-expected increase in controlling or stressful parenting (relative to prior levels) was linked to a higher predicted risk. Preschool family interaction (fli_Zm7) and its residualized change over time (change_fli_Zm7on9) also meaningfully contributed, whereas gender and changes in school adjustment had minimal importance in this model.

The SHAP summary (Figure 4) mirrors these patterns. Variables related to EF exhibited the largest absolute SHAP values (≈0.47–0.48), followed by sleep, media use, gaming proportion, and maternal-stress indicators. Positive SHAP values indicated feature effects that increased the predicted probability of persistent high risk (e.g., lower EF, shorter sleep, heavier media use, and higher gaming share), whereas higher learning-oriented media use (midUse_learn_Zc9) and longer sleep duration were associated with a lower predicted risk. Taken together, these findings suggest that early EF capacity, developmental residual change, deviations in media use, and parenting stress jointly account for a substantial proportion of the model’s predictions of persistent high-risk media overdependence.

## 4. Discussion

This study longitudinally examined how multidimensional early childhood predictors shape the developmental trajectories of digital media overdependence across Grades 3–6. Using HMM, we identified five distinct pathways, including one subgroup that persistently remained at high risk, demonstrating that the developmental patterns of media overdependence are heterogeneous rather than uniform.

A very small risk-reduction subgroup (0.7%) was also observed. Because of its small size and susceptibility to classification uncertainty, this subgroup was interpreted descriptively rather than inferentially. Nonetheless, its presence underscores the fact that not all trajectories escalate or are stable, and a small minority of children show meaningful improvement.

Difficulties in EF at Grade 1 along with further deterioration through Grade 2 were associated with remaining in the high-risk pathway. This pattern reflects a declining self-regulatory capacity and aligns with established evidence linking EF to impulsivity, attention dysregulation, and problematic media use (Diamond, 2013; Fitzpatrick et al., 2025). Early EF vulnerabilities may accumulate and increase sensitivity to immediately rewarding, socially reinforced digital stimuli. These cascading processes may include sleep disruptions that weaken next-day attention and inhibitory control, thereby reinforcing persistent risk.

Sleep has also been identified as a protective factor. Longer sleep in early childhood and increased sleep duration from preschool to Grade 1 were associated with a reduced risk. This aligns with longitudinal evidence showing that early childhood sleep problems predict later emotional and behavioral difficulties (Sivertsen et al., 2015; Quach et al., 2018). Prebedtime screen use can disrupt sleep physiology, thereby weakening next-day regulatory capacity and reinforcing media-seeking behaviors. These findings highlight sleep as a modifiable biobehavioral target with substantial implications for prevention.

Maternal parenting stress during early childhood predicts persistent high-risk pathways. Elevated stress may increase technoference, reduce behavioral consistency, and increase children’s externalizing behaviors in bidirectional cycles (McDaniel & Radesky, 2018). Conversely, consistent parental rules and strong family interactions served as protective factors. These results align with the evidence that parental monitoring and emotionally supportive relationships reduce problematic media use (Gentile et al., 2014; Lin et al., 2023). Therefore, interventions that reduce parenting stress and strengthen parent–child engagement may provide meaningful leverage points.

The proportion of game-centered media use functioned as a distinct risk factor, even after accounting for total exposure time, indicating that the structural characteristics of gaming, such as rapid reward cycles, variable reinforcement schedules, competitive ranking, and persistent progression, independently sustained overdependence risk. These dynamics may be amplified in the Korean context where gaming is deeply embedded in peer norms and social routines.

The HMM entropy indicated moderate uncertainty in state assignments, consistent with the probabilistic nature of latent-state modeling. Although machine learning analyses relied on iterbi-decoded labels, the focus on multiyear persistent patterns reduced sensitivity to wave-level misclassification. Nevertheless, developmental processes are complex and observational data cannot fully establish causality; thus, the findings should be interpreted with caution.

Methodologically, this study demonstrates the value of combining the HMM with machine learning. The HMM enables the data-driven identification of latent states without prespecifying profiles, whereas machine learning models leverage nonlinear interactions across multidomain predictors. As shown in Supplementary Table S6, XGBoost outperformed the logistic regression and random forest models in terms of the ROC AUC, F1, and PR AUC metrics, supporting its selection as the primary predictive model. The SHAP analyses further clarified the individual and joint contributions of the predictors. As demonstrated in Supplementary Table S9 and Figures S5 and S6, the SHAP interaction patterns reflected predictive— not-causal relationships and illustrated how feature pairs jointly influenced the model outputs.

Theoretically, these findings align with and extend the Interactional Theory of Childhood Problematic Media Use (IT-CPMU). The persistent high-risk trajectory and its early predictors reflect the theory’s emphasis on the interactions between self-regulatory vulnerabilities and environmental stressors. The identification of modifiable protective factors, such as sleep trajectories and consistent parental regulation, further reinforces the hypothesis that developmental pathways are malleable rather than fixed. Thus, the present findings empirically support the theoretical hypothesis that accumulated early self-regulatory and environmental risks contribute to the development of persistent high-risk media use pathways.

This study has some limitations. First, the measures were not designed to test the hypotheses. Second, some variables relied on parental reports, which may have been susceptible to recall or perception biases. Third, moderate state uncertainty may have contributed to the borderline classifications. Fourth, machine learning models inherit this uncertainty from the decoded labels. Finally, predictive models support associations rather than causal inferences. Future research should incorporate multiple informant reports, objective assessments, and causal inference designs to strengthen precision and validity.

## 5. Conclusions

This study identified modifiable early life factors associated with persistent digital media overdependence. Despite the moderate state uncertainty in the HMM, one high-risk trajectory remained stable across all four waves. EF difficulties in Grade 1 and their deterioration through Grade 2 increased the likelihood of remaining in this persistent-risk group. In contrast, sufficient early childhood sleep, an upward sleep trajectory, consistent parental rules, and strong family interactions served as protective factors. Higher maternal parenting stress, longer total media time, and a greater proportion of game-centered media use were important risk factors. Combining HMMs with machine learning enhances both the interpretability of developmental pathways and the practical value of predictive modeling.

In practice, key priorities include supporting EF and self-regulation during the early elementary years, promoting healthy sleep routines, identifying and reducing parenting stress, strengthening consistent household rules and positive family interactions, and improving parent–child literacy regarding structural risks common in game-based media. Future research should refine early indicators that differentiate acceleration from stable risk, incorporate objective usage and contextual data, and evaluate fairness and generalizability across diverse cultural settings so that predictive insights can support evidence-based practices in homes and schools.

## Figures and Tables

**Table 1:** Changes in Media Overdependence Scores Across User Risk Groups (*n* = 1354).

Wave	Variable	Total		General Users			Potential Risk Users			High Risk Users			*F*	*p*
		*M*	*SD*	*M*	*SD*	%	*M*	*SD*	%	*M*	*SD*	%		
w10	Total Score	23.59	5.94	20.87	3.32	76.11	28.42	0.64	5.78	33.42	3.61	18.11	1452.19	<0.001
	F1	7.22	2.13	6.28	1.16		8.78	1.19		10.66	1.57		1217.62	<0.001
	F3	7.03	1.88	6.36	1.45		8.18	1.08		9.45	1.45		464.26	<0.001
	F4	5.89	1.75	5.24	1.26		6.95	1.10		8.27	1.41		550.44	<0.001
w11	Total Score	24.57	6.31	20.97	3.27	68.31	28.35	0.67	7.05	33.47	3.51	24.64	1866.55	<0.001
	F1	7.63	2.33	6.37	1.22		8.60	1.23		10.85	1.57		1405.66	<0.001
	F3	7.13	1.93	6.26	1.43		8.24	1.14		9.25	1.46		564.02	<0.001
	F4	6.27	1.86	5.40	1.29		7.16	1.36		8.42	1.37		657.73	<0.001
w12	Total Score	26.26	6.35	21.53	3.46	55.78	28.41	0.74	9.36	33.26	3.20	34.85	1842.65	<0.001
	F1	8.32	2.32	6.68	1.34		9.00	1.19		10.78	1.28		1365.16	<0.000
	F3	7.51	1.98	6.31	1.47		8.20	1.29		9.26	1.36		606.35	<0.000
	F4	6.75	1.88	5.58	1.28		7.25	1.19		8.47	1.38		673.80	<0.000
w13	Total Score	27.24	6.52	22.10	3.19	52.32	28.24	0.78	9.81	34.09	3.78	37.87	1925.98	<0.000
	F1	8.41	2.47	6.58	1.35		8.61	1.31		10.88	1.58		1296.91	<0.000
	F3	7.75	1.98	6.42	1.34		8.14	1.08		9.49	1.42		753.06	<0.000
	F4	7.30	1.91	6.10	1.35		7.56	1.30		8.89	1.46		586.77	<0.000

*Note.* Analyses were restricted to participants who responded to three or more waves. Wave-specific missing rates were W10 = 5.4%, W11 = 2.65%, W12 = 6.1%, and W13 = 2.9%. Subscale definitions: F1 = Self-regulation difficulties, F3 = Disturbance in daily life, F4 = Control failure. Each subdomain (self-regulation difficulty, daily life interference, and control failure) also showed a significant increase over time (all *p* < 0.001). The proportion of general users decreased from 76.1% at w10 to 52.3% at w13, while the proportion of high-risk users increased from 18.1% to 37.9%, and that of at-risk users increased from 5.8% to 9.8%. Taken together, these results indicate that within the analytical sample of the present study, measured levels of media overdependence and the proportion classified as high-risk increased over the observed period.

**Table 2:** Model fit statistics for competing HMMs.

Model	No_of_States	LogLik	AIC	BIC	Entropy	Converged
HMM-2	2	−3893.32	7800.64	7846.51	0.72	TRUE
HMM-3	3	−3856.17	7740.34	7832.09	0.56	TRUE
HMM-4	4	−3846.56	7739.12	7889.86	0.63	TRUE

**Table 3:** Transition probability matrix (HMM-3).

From	To_General (S1)	To_Potential Risk (S2)	To_High Risk (S3)
General	0.750	0.250	0.000
Potential Risk	0.118	0.561	0.321
High Risk	0.013	0.091	0.897

**Table 4:** Predictors Entered into the Machine Learning Model.

Variable	Overall		Min	Max	High-Risk		Non–High-Risk		*t*/χ^2^	*p*
	*M/n*	*SD/%*			*M/n*	*SD/%*	*M/n*	*SD/%*		
gender_c	686	50.66			118	62.77	568	48.71	12.79	<0.001
age_y_c10	9.38	0.12	9.17	9.75	9.39	0.13	9.38	0.11	1.55	0.122
age_m10	39.80	3.64	28.00	55.00	39.87	4.00	39.79	3.58	0.30	0.766
age_f10	42.25	3.92	28.00	59.00	42.44	4.11	42.21	3.89	0.74	0.462
edu_m10_college_or_higher	991	73.19			121	64.36	870	74.61	8.67	0.004
edu_f10_college_or_higher	993	73.34			134	71.28	859	73.67	0.48	0.479
income_m_h10	534.23	441.69	90.00	8500.00	532.28	471.90	534.54	436.83	−0.07	0.948
cbt_expT_c7	45.64	9.53	31.00	90.00	49.95	10.44	44.94	9.20	6.79	<0.001
sleepTime_c7	9.76	0.69	7.00	12.50	9.55	0.74	9.80	0.68	−4.56	<0.001
change_sleepTime_Zc9on78	0.00	0.88	−2.66	4.11	−0.09	0.98	0.02	0.87	−1.60	0.110
exf_c8	1.44	0.31	1.00	3.00	1.62	0.36	1.41	0.29	7.77	<0.001
change_exf_Zc8on9	0.01	0.71	−3.15	2.86	0.28	0.88	−0.04	0.67	4.74	<0.001
pScl_c8	4.44	0.65	1.00	5.00	4.22	0.74	4.47	0.63	−4.99	<0.001
change_pScl_Zc8on9	−0.01	0.92	−3.64	2.87	−0.14	0.98	0.01	0.91	−2.09	<0.001
midUseTime_c8	0.40	0.49	0.00	5.00	0.57	0.59	0.37	0.47	4.56	<0.001
change_midUseTime_Zc8on9	0.00	0.94	−2.25	5.41	0.26	1.21	−0.04	0.88	3.21	0.002
midUse_game_c9	18.87	29.22	0.00	100.00	48.04	17.19	32.65	31.34	6.54	<0.001
midUse_learn_c9	34.79	31.55	0.00	100.00	9.17	29.69	20.43	30.44	−7.33	<0.001
prs_m7	2.57	0.63	1.00	4.73	2.86	0.58	2.52	0.62	6.91	<0.001
change_prs_Zm7on8	0.01	0.76	−3.40	2.98	0.17	0.71	−0.02	0.77	3.21	0.001
fli_m7	3.78	0.53	1.00	5.00	3.58	0.53	3.81	0.52	−5.74	<0.001
change_fli_Zm7on9	−0.01	0.81	−3.86	5.02	−0.18	0.86	0.02	0.79	−3.25	0.001
crs_ct_m7	3.46	0.48	1.00	5.00	3.46	0.40	3.47	0.50	−0.17	0.868
change_crs_ct_Zm7on8	−0.01	0.83	−5.18	3.47	−0.14	0.70	0.01	0.84	−2.31	0.021
crs_ct_f7	3.31	0.56	1.00	5.00	3.28	0.54	3.32	0.57	−0.81	0.420
change_crs_ct_Zf7on8	0.00	0.84	−4.18	3.60	−0.13	0.85	0.03	0.83	−2.39	0.017

*Notes*. Owing to space constraints, the left-hand category column was omitted. Variable groups are: Baseline (age-9) demographics = gender_c, age_y_c10, age_m10, age_f10, edu_m10_college_or_higher, edu_f10_college_or_higher, income_m_h10; Child factors = cbt_expT_c7, sleepTime_c7, change_sleepTime_Zc9on78, exf_c8, change_exf_Zc8on9, pScl_c8, change_pScl_Zc8on9, midUseTime_c8, change_midUseTime_Zc8on9, midUse_game_c9, midUse_learn_c9; Parent factors = prs_m7, change_prs_Zm7on8, fli_m7, change_fli_Zm7on9, crs_ct_m7, change_crs_ct_Zm7on8, crs_ct_f7, change_crs_ct_Zf7on8. Variables prefixed with “change_” denote residualized change (the residual from regressing the later value on the prior value; e.g., Grade-2 on Grade-1). Positive values indicate an increase relative to their peers at the same level. *M*/*n* and *SD*/% indicate the mean and standard deviation for continuous variables and counts/percentages for categorical variables, respectively. “High-Risk” refers to the consistently high-risk group; “Non–High-Risk” includes all others. Time point tags: c7, early childhood, c8 = Grade 1, c9 = Grade 2, c10 = age-9 baseline (demographics).

**Table 5:** Summary of Model Predictive Performance (Test Set).

Metric	Estimate (95% CI)
ROC AUC (DeLong)	0.84 (0.78–0.91)
Sensitivity	0.82 (0.63–0.94)
Specificity	0.71 (0.63–0.77)
PPV	0.31 (0.21–0.43)
NPV	0.96 (0.91–0.99)
F1-score	0.45 (0.33–0.56)
PR AUC	0.48 (0.29–0.65)
Brier score	0.13
ECE	0.15 (0.11–0.19)
MCE	0.57 (0.44–0.82)

*Note.* The 95% confidence intervals (CIs) were calculated using 2000 bootstrap re-samples or the DeLong method. The hyperparameters, confusion matrix/summary, and PR curves are provided in Supplementary Tables S7, S8, and Figure S4.

## Data Availability

The datasets generated and/or analyzed in the current study are available in the Panel Study on Korean Children repository, which is available online at https://panel.kicce.re.kr/pskc/index.do (accessed 9 June 2025).

## Supplementary Materials

Supporting information can be downloaded from: https://www.mdpi.com/article/10.3390/bs15121725/s1, Table S1. Variable Code Definitions. Table S2. Percentage of Missing Values for Each Variable. Table S3. Classification of Developmental Trajectory Groups. Table S4. Emission (response) probability for each latent state. Table S5. Transition probabilities and 95% bootstrap confidence interval latent states. Table S6. Comparative performances of XGBoost, logistic regression, and random forest classifiers. Table S7. XGBoost Model Hyperparameter Settings. Table S8. Confusion Matrix and Basic Classification Summary. Table S9. Top 20 SHAP Interaction Effects from the XGBoost Model. Figure S1: Correlation Matrix (Training Data, Pearson’s *r*); Figure S2: Trace Plots Showing Convergence of MICE Imputation Chains; Figure S3: Distributional Comparison Across Imputed Datasets; Figure S4: Precision–Recall (PR) Curve Showing Class Imbalance Performance; Figure S5: SHAP Interaction Plot: Executive Function × Change in Sleep Duration; Figure S6: SHAP Interaction Plot: Gaming Proportion × Total Media Time.
