# Supplementary material for: Hidden Markov Trajectories of Early-Adolescent Media Overdependence and Machine Learning Prediction of High-Risk Maintenance from Early Childhood and Lower Elementary Predictors
Source: Behav Sci (Basel). 2025 Dec 12;15(12):1725. doi: 10.3390/bs15121725 (PMC12729965; doi:10.3390/bs15121725)
Supplement: Supplementary file 1 [file behavsci-15-01725-s001.zip › (Revised) Supplementary_Table.pdf]

Supplementary Table S1. Variable Code Definitions

Variable Code	Label
gender_c	Sex (child)
age_y_c10	Child age (years), wave 10
age_m10	Mother's age, wave 10
age_f10	Father's age, wave 10
edu_univ_m10	Mother: university degree (wave 10)
edu_univ_f10	Father: university degree (wave 10)
income_m_h10	Household income (monthly, wave 10)
cbt_expT_c7	Externalizing problems (age 6y; w7)
sleepTime_c7	Sleep duration (age 6y; w7)
change_sleepTime_Zc9on78	Change in sleep duration to age 8y (residualized on ages 6y and 7y; w9 on w7–w8)
exf_c8	Executive function (age 7y; Grade 1; w8)
change_exf_Zc8on9	Change in executive function (age 7y→8y; Grade 1→2; w8→w9)
pScl_c8	School adjustment (age 7y; Grade 1; w8)
change_pScl_Zc8on9	Change in school adjustment (age 7y→8y; Grade 1→2; w8→w9)
midUseTime_c8	Media use time (age 7y; Grade 1; w8)
change_midUseTime_Zc8on9	Change in media use time (age 7y→8y; Grade 1→2; w8→w9)
midUse_game_c9	Media use – gaming proportion (%) (age 8y; Grade 2; w9)
midUse_learn_c9	Media use – learning proportion (%) (age 8y; Grade 2; w9)
prs_m7	Maternal parenting stress (age 6y; w7)
change_prs_Zm7on8	Change in maternal parenting stress (age 6y→7y; w7→w8)
fli_m7	Maternal family interaction (age 6y; w7)
change_fli_Zm7on9	Change in maternal family interaction (age 6y→8y; w7→w9)
crs_ct_m7	Maternal controlling parenting (age 6y; w7)
change_crs_ct_Zm7on8	Change in maternal controlling parenting (age 6y→7y; w7→w8)
crs_ct_f7	Paternal controlling parenting (age 6y; w7)
change_crs_ct_Zf7on8	Change in paternal controlling parenting (age 6y→7y; w7→w8)

Note.
Korean school system: In Korea, age 7 years = Grade 1 (w8) and age 8 years = Grade 2 (w9)

Variables with the prefix *change_* represent residualized change scores, controlling for the corresponding baseline values.

Supplementary Table S2. Percentage of Missing Values for Each Variable

Variable	(%)
midUse_learn_c9	16.03
midUse_game_c9	15.95
change_crs_ct_Zf7on8	14.48
income_m_h10	11.15
change_fli_Zm7on9	9.75
crs_ct_f7	9.53

Variable	(%)
change_exf_Zc8on9	8.71
change_prs_Zm7on8	7.68
change_crs_ct_Zm7on8	7.68
fli_m7	5.47
prs_m7	5.47
crs_ct_m7	5.47
change_sleepTime_Zc9on78	5.47
exf_c8	4.80
change_pScl_Zc8on9	4.58
change_midUseTime_Zc8on9	4.43
cbt_expT_c7	3.69
income_m_h7	3.32
age_y_c10	3.25
pScl_c8	3.03
sleepTime_c7	2.95
midUseTime_c8	2.88
edu_univ_m10	2.29
age_f10	2.14
edu_univ_f10	2.14
age_m10	2.07
Media overdependence (wave 10)	0.05
Media overdependence (wave 11)	2.6
Media overdependence (wave 12)	6.1
Media overdependence (wave 13)	2.9

Note.
The percentages indicate the proportion of missing values for each variable. The rows labeled "Media overdependence (wave 10–13)" represent missing rates across waves for the dependent variable (*media overdependence*).

***Supplementary Table S3.* Classification of Developmental Trajectory Groups**

Trajectory Group	*n*	%	Typical Path
Group 1: Stable Normal	529	39.1	Normal → Normal → Normal → Normal
Group 2: Consistently High-Risk	188	13.9	High-Risk → High-Risk → High-Risk → High-Risk
Group 3: Gradually Increasing Risk	242	17.9	Normal → At-Risk → High-Risk → High-Risk
Group 4: Risk Reduction (from High-Risk)	10	0.7	High-Risk → High-Risk → At-Risk → At-Risk
Group 5: Fluctuating	385	28.4	Normal → Normal → Normal → At-Risk

Note.
The table presents the classification of developmental trajectory groups identified through posterior decoding. Group 1 (*Stable Normal*) indicates children who consistently maintained a normal developmental level; Group 2 (*Consistently High-Risk*) includes children who remained in the high-risk range across all waves; Group 3 (*Gradually Increasing Risk*) represents children who showed a progressive increase in risk status from normal to high-risk; Group 4 (*Risk Reduction*) refers to children who initially exhibited high-risk status but later improved to at-risk levels; Group 5 (*Fluctuating*) describes children who generally maintained a normal pattern but ended with an at-risk level, indicating instability over time.

***Supplementary Table S4.* Emission (Response) Probabilities for Each Latent State**

State	$P(Y = \text{Normal})$	$P(Y = \text{At-Risk})$	$P(Y = \text{High-Risk})$
Normal	0.953	0.017	0.030
At-Risk	0.433	0.244	0.323
High-Risk	0.073	0.038	0.889

Note.
P denotes probability, and Y represents the observed response variable indicating the child's developmental status (Normal, At-Risk, or High-Risk).
The table presents the emission (response) probabilities estimated for each latent state in the hidden Markov model; the *Normal* latent state shows a high probability of responding as normal, indicating strong state stability; the *At-Risk* state exhibits moderate probabilities across categories, reflecting transitional or mixed characteristics; the *High-Risk* state shows a very high probability of responding as high-risk, suggesting high internal consistency within this latent class.

***Supplementary Table S5.* Transition probabilities and 95% bootstrap confidence intervals latent states**

From state	To state	Estimate	95% CI lower	95% CI upper
Normal	Normal	0.748	0.730	0.764
Normal	At-risk	0.085	0.075	0.096
Normal	High-risk	0.167	0.151	0.184
At-risk	Normal	0.414	0.362	0.477
At-risk	At-risk	0.121	0.084	0.159
At-risk	High-risk	0.465	0.411	0.516
High-risk	Normal	0.245	0.215	0.279
High-risk	At-risk	0.084	0.066	0.100
High-risk	High-risk	0.671	0.633	0.706

Note.
Estimates represent latent-state transition probabilities based on a 3-state Hidden Markov Model. 95% confidence intervals were computed using 500 bootstrap samples, resampled at the participant level.

***Supplementary Table S6.* Comparative Performance of XGBoost, Logistic Regression, and Random Forest Classifiers**

Model	ROC AUC	F1	PR AUC
XGBoost	0.844	0.451	0.476
Logistic	0.816	0.471	0.441
RandomForest	0.836	0.433	0.459

Note.
Across all evaluated metrics (AUC, F1, and AUPRC), XGBoost demonstrated the most favorable balance between discrimination and precision–recall performance. In particular, the model achieved the highest AUPRC (0.476), which is critical for imbalanced outcomes such as the high-risk trajectory in this study. Although logistic regression yielded a slightly higher F1 score, XGBoost offered the strongest overall performance and stability, supporting its selection as the final predictive model.

Supplementary Table S7. **XGBoost Model Hyperparameter Settings**

Parameter	Candidate Values (Grid)	Selected Value (bestTune)	Description
nrounds	200	200	Number of boosting iterations.
max_depth	3, 5	3	Maximum depth of individual trees.
eta	0.05, 0.1	0.05	Learning rate (step size shrinkage).
gamma	0	0	Minimum loss reduction required to make a further partition on a leaf node.
colsample_bytree	0.8	0.8	Subsampling ratio of columns per tree.
min_child_weight	1, 5	5	Minimum sum of instance weights needed in a child node.
subsample	0.8	0.8	Subsampling ratio of the training instances.
Cross-validation	3-fold stratified	3 folds	Internal model selection procedure (stratified 3-fold CV).
Performance metric	ROC (AUC)	–	Optimization target: maximize the area under the ROC curve.
Preprocessing	Median imputation, centering, scaling	–	Data preprocessing applied prior to model training.

Notes.

The XGBoost classifier was optimized using a stratified 3-fold cross-validation procedure, targeting the highest AUC. All input features were preprocessed using median imputation for missing values, followed by centering and scaling to standardize the feature space.

Supplementary Table S8. **Confusion Matrix and Basic Classification Summary**

Threshold	TP	FP	TN	FN	Accuracy (95% CI)	Prevalence
0.2954	23	51	123	5	0.72 (0.66–0.78)	0.14

Note.
This table summarizes the model's binary classification performance at the optimal threshold (0.2954); TP (true positives) = 23 cases correctly predicted as positive; FP (false positives) = 51 cases incorrectly predicted as positive; TN (true negatives) = 123 cases correctly predicted as negative; FN (false negatives) = 5 cases incorrectly predicted as negative. The model achieved an accuracy of 0.72 (95% CI = 0.66–0.78), with a prevalence of 0.14 in the dataset.

Supplementary Table S9. **Top 20 SHAP Interaction Effects from the XGBoost Model**

feature_1	feature_2	interaction_score
exf_Zc8	change_exf_Zc8on9	0.202435
exf_Zc8	midUseTime_Zc8	0.140125
change_exf_Zc8on9	midUseTime_Zc8	0.124464
prs_Zm7	exf_Zc8	0.115736
exf_Zc8	midUse_game_Zc9	0.111908
sleepTime_Zc7	exf_Zc8	0.111864
midUse_game_Zc9	change_exf_Zc8on9	0.109058
prs_Zm7	change_exf_Zc8on9	0.107917
sleepTime_Zc7	change_exf_Zc8on9	0.102646
exf_Zc8	change_crs_ct_Zm7on8	0.100204
change_crs_ct_Zm7on8	change_exf_Zc8on9	0.095094
change_crs_ct_Zf7on8	change_exf_Zc8on9	0.089029
midUse_learn_Zc9	change_exf_Zc8on9	0.078982
sleepTime_Zc7	midUseTime_Zc8	0.078966
exf_Zc8	change_crs_ct_Zf7on8	0.07859
midUse_game_Zc9	midUseTime_Zc8	0.076987
exf_Zc8	midUse_learn_Zc9	0.075647
exf_Zc8	change_midUseTime_Zc8on9	0.073657
prs_Zm7	midUseTime_Zc8	0.069891
fli_Zm7	exf_Zc8	0.067913

Note.

This table presents the 20 strongest pairwise SHAP interaction effects derived from the XGBoost classifier. Higher interaction values indicate a stronger combined contribution of the corresponding feature pair to the model's prediction of the high-risk trajectory.
