# Supplementary material for: Hidden Markov Trajectories of Early-Adolescent Media Overdependence and Machine Learning Prediction of High-Risk Maintenance from Early Childhood and Lower Elementary Predictors
Source: Behav Sci (Basel). 2025 Dec 12;15(12):1725. doi: 10.3390/bs15121725 (PMC12729965; doi:10.3390/bs15121725)
Supplement: Supplementary file 1 [file behavsci-15-01725-s001.zip › (Revised) Supplementary_Figure.pdf]

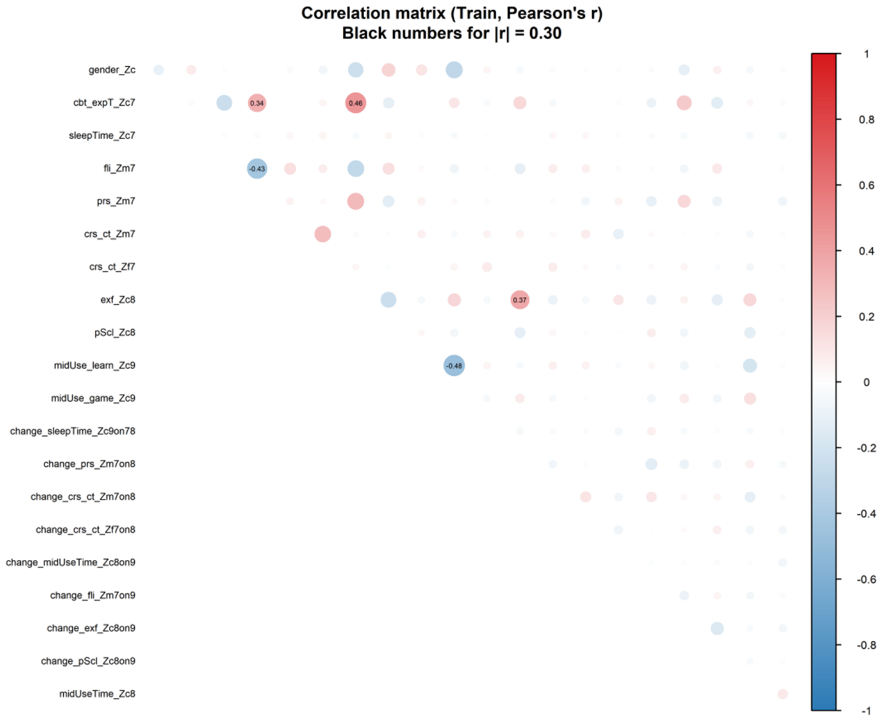

Supplementary Figure S1. **Correlation Matrix (Training Data, Pearson's *r*)**

Note.
The figure presents a Pearson correlation matrix displaying associations among predictor variables in the training dataset.
Circles indicate the direction and magnitude of correlations (red = positive; blue = negative); black numbers denote coefficients with $|r| \geq .30$.Pairs of variables showing correlations with $|r| \geq .30$ were as follows: *midUse_learn_Zc9* and *midUse_game_Zc9* ($r = -.478$); *cbt_expT_Zc7* and *exf_Zc8* ($r = .455$); *fli_Zm7* and *prs_Zm7* ($r = -.429$); *exf_Zc8* and *change_prs_Zm7on8* ($r = .372$); *cbt_expT_Zc7* and *prs_Zm7* ($r = .343$). These correlations indicate that all predictors used in the machine learning model show absolute correlations below .50.

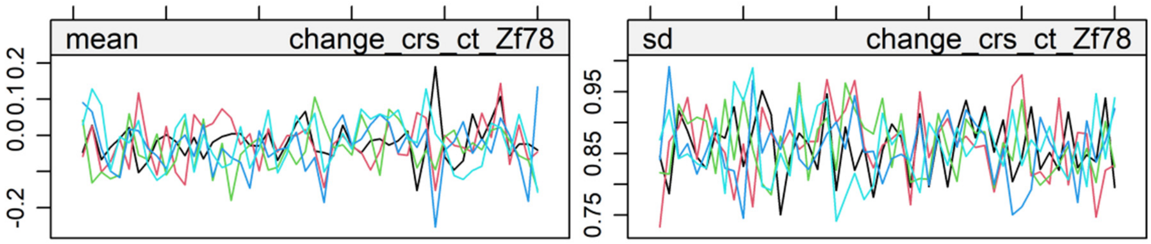

Supplementary Figure S2. **Trace Plots Showing Convergence of MICE Imputation Chains**

Note.
Supplementary Figure S2. Trace plots of means and standard deviations for the imputed variable change_crs_ct_Zf78 across 50 MICE iterations.

The overlaid chains indicate stable mixing and no visible trends, supporting adequate convergence of the imputation process.

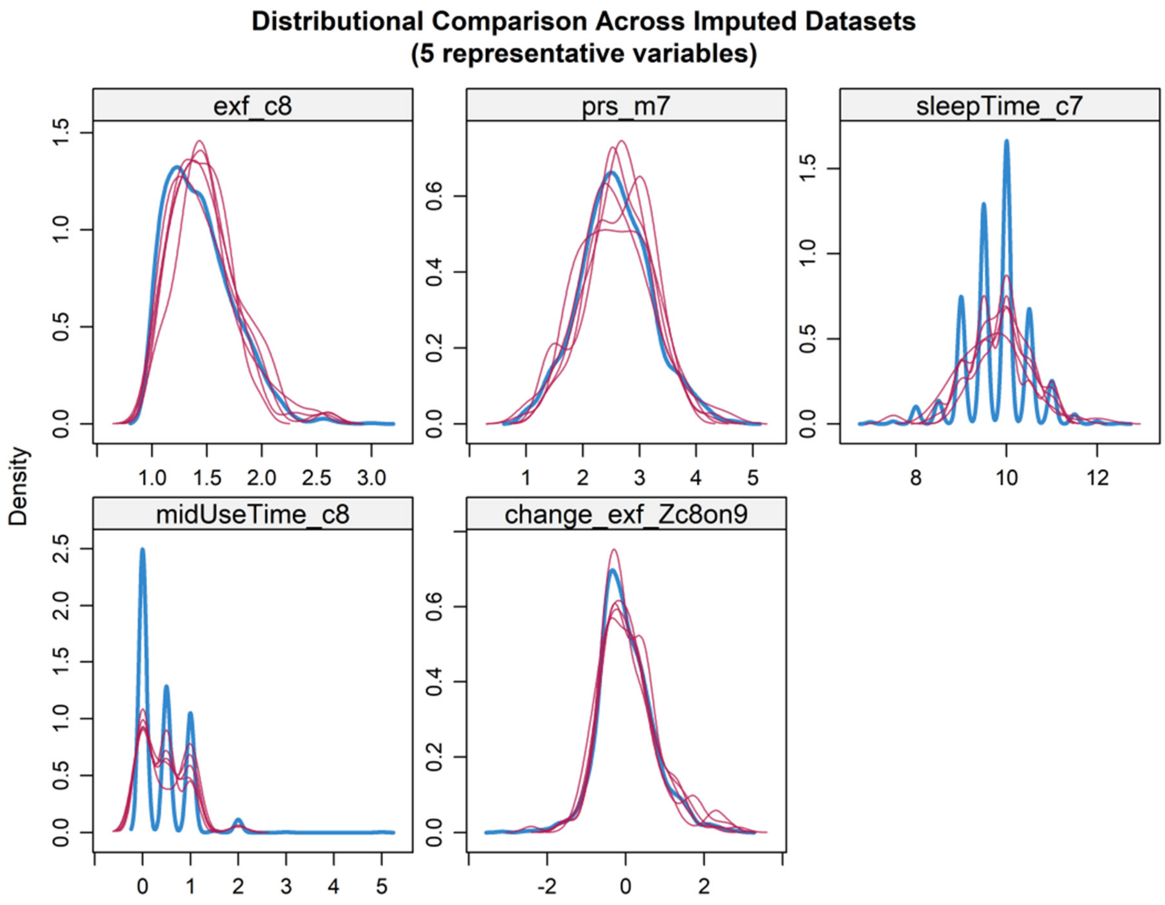

Supplementary Figure S3. **Distributional Comparison Across Imputed Datasets**

Note.

Supplementary Figure S3. Kernel density plots comparing the distributions of key analytic variables (exf_c8, prs_m7, sleepTime_c7, midUseTime_c8, change_exf_Zc8on9) across all imputed datasets.

The highly overlapping densities indicate that the imputation procedure preserved the original distributional structure without introducing systematic bias.

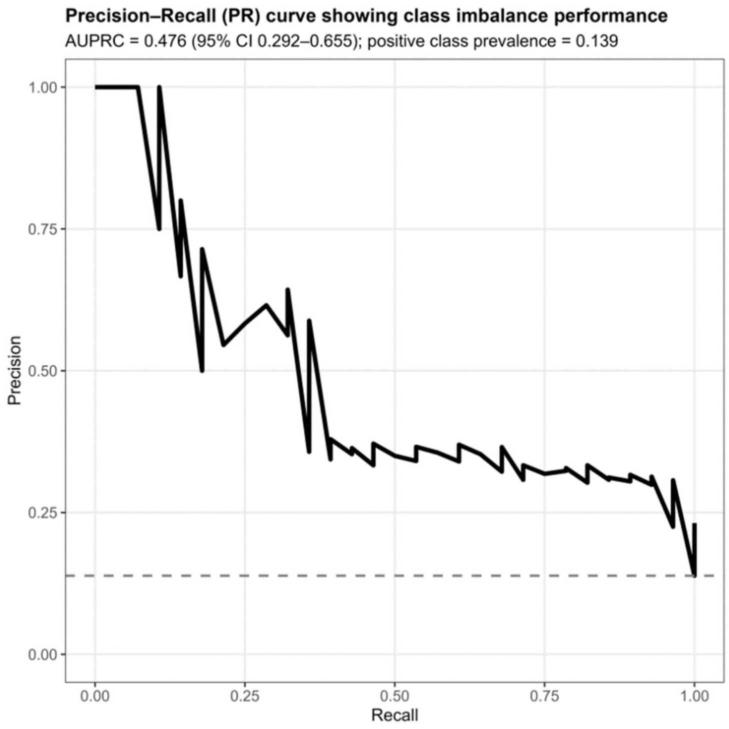

Supplementary Figure S4. **Precision–Recall (PR) Curve Showing Class Imbalance Performance**

Note.

The PR curve illustrates the XGBoost model's performance under class imbalance conditions.
The area under the precision–recall curve (AUPRC) was 0.48 (95% CI, 0.29–0.65), indicating stable predictive performance even with low positive case prevalence.

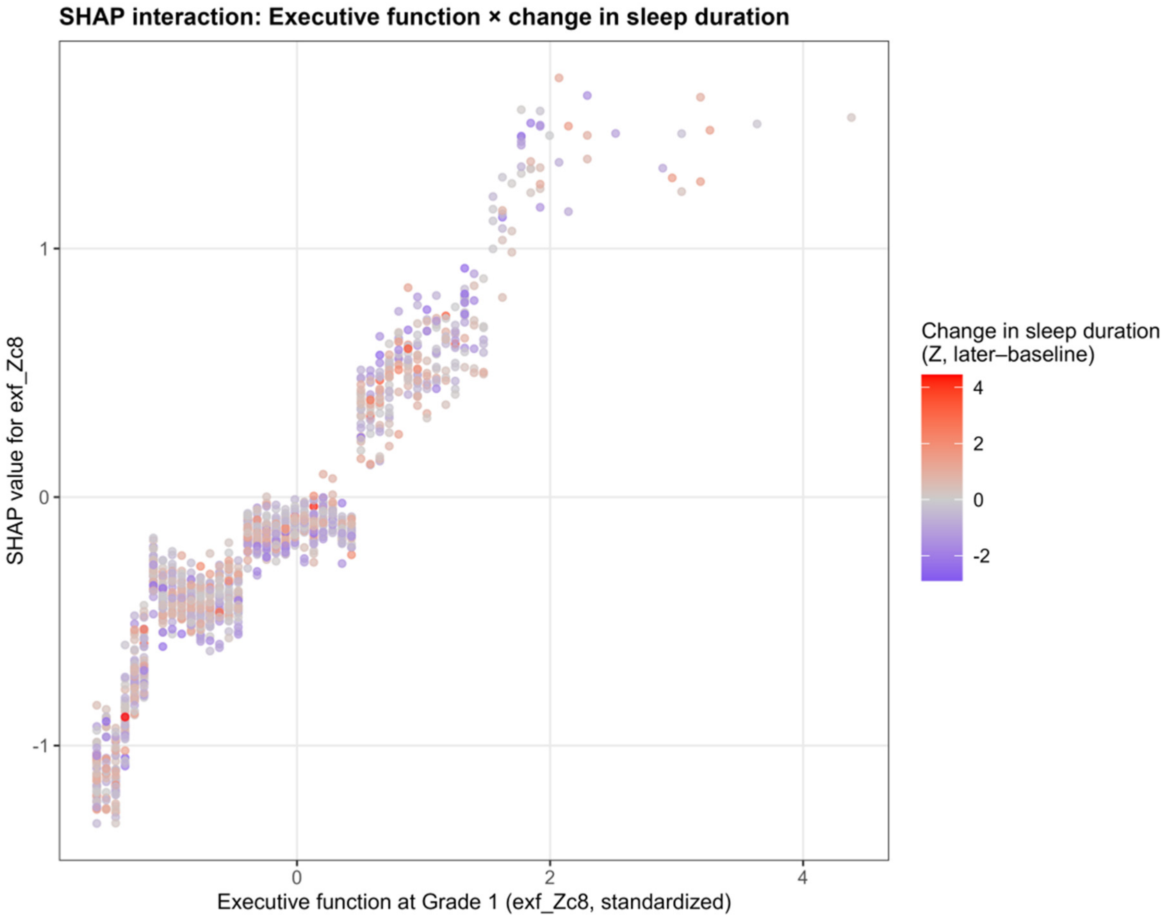

Supplementary Figure S5. **SHAP Interaction Plot: Executive Function × Change in Sleep Duration**

Note.

The plot visualizes how executive function (exf_Zc8) interacts with change in sleep duration (change_sleepTime_Zc9on78) to influence the predicted risk.

Children with lower EF and concurrent reductions in sleep duration show sharply increased SHAP values, indicating a heightened contribution to the high-risk trajectory classification.

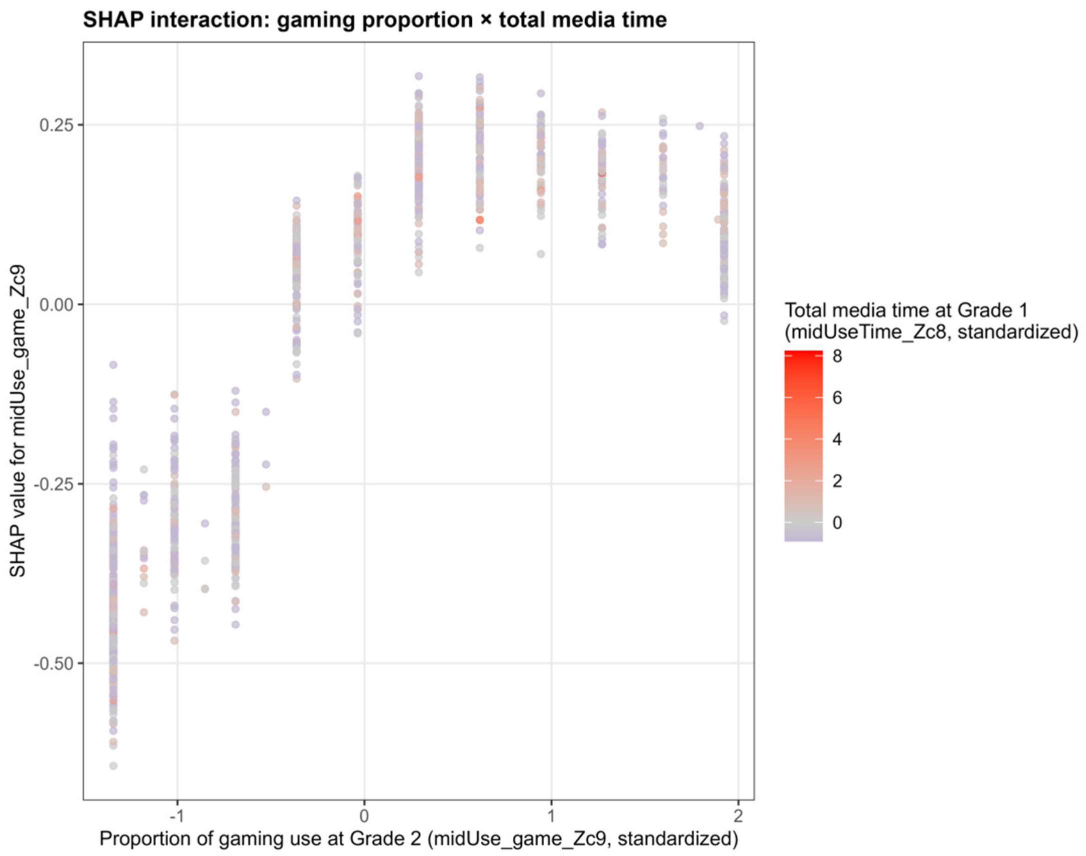

Supplementary Figure S6. **SHAP Interaction Plot: Gaming Proportion × Total Media Time**

Note.

This figure shows the interaction between the proportion of gaming use (midUse_game_Zc9) and total media time (midUseTime_Zc8).

Gaming intensity amplifies the risk effect of total media time, revealing a synergistic pattern such that high gaming proportion combined with high total media exposure produces elevated SHAP values associated with the high-risk trajectory.
